# Assessment of genomic changes in a CRISPR/Cas9 *Phaeodactylum tricornutum* mutant through whole genome resequencing

**DOI:** 10.7717/peerj.5507

**Published:** 2018-10-05

**Authors:** Monia Teresa Russo, Riccardo Aiese Cigliano, Walter Sanseverino, Maria Immacolata Ferrante

**Affiliations:** 1 Department of Integrative Marine Ecology, Stazione Zoologica Anton Dohrn, Naples, Italy; 2 Sequentia Biotech, Bellaterra, Spain

**Keywords:** CRISPR/Cas9, Diatoms, Off-target, Bacterial conjugation, Genome editing, Loss of heterozygosity, Knock-out, Whole genome resequencing

## Abstract

The clustered regularly interspaced short palindromic repeat (CRISPR)/Cas9 system, co-opted from a bacterial defense natural mechanism, is the cutting edge technology to carry out genome editing in a revolutionary fashion. It has been shown to work in many different model organisms, from human to microbes, including two diatom species, *Phaeodactylum tricornutum* and *Thalassiosira pseudonana*. Transforming *P. tricornutum* by bacterial conjugation, we have performed CRISPR/Cas9-based mutagenesis delivering the nuclease as an episome; this allowed for avoiding unwanted perturbations due to random integration in the genome and for excluding the Cas9 activity when it was no longer required, reducing the probability of obtaining off-target mutations, a major drawback of the technology. Since there are no reports on off-target occurrence at the genome level in microalgae, we performed whole-genome Illumina sequencing and found a number of different unspecific changes in both the wild type and mutant strains, while we did not observe any preferential mutation in the genomic regions in which off-targets were predicted. Our results confirm that the CRISPR/Cas9 technology can be efficiently applied to diatoms, showing that the choice of the conjugation method is advantageous for minimizing unwanted changes in the genome of *P. tricornutum*.

## Introduction

Diatoms are photosynthetic unicellular eukaryotes responsible for 20% of the global carbon fixation and present in marine and freshwater habitats with roughly 100,000 different species, all characterized by the presence of a silica shell and extraordinary richness of different cell shapes (Falkowski, Barber & Smetacek, 1998; Armbrust, 2009; Mann & Vanormelingen, 2013). These organisms have been attracting scientific interest for a long time for their ecological role and extraordinary biodiversity, for their complex evolutionary history and physiological properties, for the capability of adaptation to considerably diverse environments thanks to unexpected metabolic abilities, and, more recently, for their production of biotechnologically valuable products (Kroth, 2007; Kröger, 2007; Bozarth, Maier & Zauner, 2009; Archibald, 2015). Several diatom genomes have been sequenced in the last few years. Their comparison has revealed that a large amount of the genes are not shared among different species and, moreover, do not have a predictable function when comparing with existing databases. In order to unveil the molecular mechanisms that are at the basis of diatom biology, it is crucial to enrich resources and tools to study and manipulate these organisms (Armbrust et al., 2004; Bowler et al., 2008; Lommer et al., 2012; Tanaka et al., 2015; Mock et al., 2017; Basu et al., 2017). Many efforts have been made in the last decade in this direction. Nowadays, it is possible to knock-down and overexpress genes of interest (De Riso et al., 2009), or to localize the protein product thanks to a fluorescent tag (Siaut et al., 2007), and besides gene expression modulation it is also possible, in *Phaeodactylum tricornutum* (Nymark et al., 2016) and in *Thalassiosira pseudonana* (Hopes et al., 2016), to permanently modify the genome obtaining knock-out or knock-in mutants by means of clustered regularly interspaced short palindromic repeats (CRISPRs).

CRISPRs are repetitive sequences found in bacterial and archaeal genomes interrupted by spacers captured from previously faced virus genomes and other invasive DNA. They provide adaptive immunity via CRISPR associated (Cas) proteins that act as RNA-directed endonucleases to degrade the same type of invasive DNA if it is encountered again (Lee et al., 2016). To date, three CRISPR/Cas subtypes have been classified (Kumar & Jain, 2015). Among them, the type II CRISPR/Cas system derived from *Streptococcus pyogenes* is the most commonly used based on its relative simplicity (Hsu, Lander & Zhang, 2014). In particular, the type II CRISPR system utilizes a single endonuclease protein Cas9 to induce DNA cleavage (Chylinski et al., 2014). This microbial defense mechanism has been co-opted to carry out mutagenesis through two components, the Cas9 nuclease and a single guide RNA (sgRNA) directing the nuclease to a specific DNA sequence, representing the target site of interest. To accomplish its function, the target site has to be located immediately upstream of a protospacer adjacent motif (PAM), a very short sequence that is recognized by the nuclease. Cleavage occurs three nucleotides upstream of the PAM on both strands, mediated by the Cas9 endonuclease introducing a precise double-strand break (DSB) with blunt ends (Chen & Gao, 2014; Doudna & Charpentier, 2014; Osakabe & Osakabe, 2015). DSB can be repaired by a highly efficient but error-prone non homologous end-joining (NHEJ) pathway that causes mutations at the breakpoint.

In diploid organisms, targeted mutations can be monoallelic or biallelic, homozygous or heterozygous, the latter resulting from the creation of two different mutant alleles at the target (Bortesi et al., 2016).

Despite the success of the CRISPR/Cas9 and the large use of the technology, due to the high efficiency and the user-friendly protocol with low costs, many drawbacks still have to be understood and overcome. In particular, the application of the technology can imply the occurrence of unwanted off-target mutations. Off-targets prediction tools have been developed; these, however, are not always reliable and some predicted off-target sites may be ignored by the enzyme while DSBs may be introduced elsewhere (Sander & Joung, 2014; Anderson et al., 2015).

We used the CRISPR/Cas9 system to edit the Phatr3_J46193 gene in the *P. tricornutum* genome transforming cells by bacterial conjugation, a method recently extended to diatoms (Karas et al., 2015). Bacterial conjugation was chosen in place of the traditional biolistic method because this latter method causes random integration of the transgene in the genome and therefore has some disadvantages, such as occurrence of unwanted perturbations of gene expression due to position effects, causing unspecific phenotypes, or frequent absence of transgene expression due to fragmentation and incomplete incorporation of the transgene. Moreover, for the CRISPR/Cas9 application, bacterial conjugation offers the great advantage for allowing the exclusion of the nuclease activity from the cells once the desirable gene modification has been obtained (Slattery et al., 2018). It is indeed largely reported in literature that off-target effects can be due to the high amount of enzyme in the cell, with continuous and uncontrollable nuclease activity (Hsu et al., 2013; Spicer & Molnar, 2018).

We analyzed the mutated strains by Sanger sequencing, observing a high mutation rate at the specific locus, but to measure the value of this approach for functional studies it is critical to examine whether it is possible to generate gene-edited cells with minimal mutational load. Allowing for quantitative and sensitive detection of targeted mutations, whole genome sequencing (WGS) is the most intuitive and comprehensive method to identify mutations induced by Cas9 at the whole genome level. Our WGS data confirmed the specific mutation and indicated a certain number of unspecific changes unlikely ascribed to nuclease activity because we observe a comparable amount of changes in the wild type and mutant strains, most likely due to in-culture evolution of the cells.

## Materials and Methods

### Target sequence design

Putative target sequences found in the locus chr9: 533409–537647 of the *P. tricornutum* genome were BLASTed against the same genome with the following criteria on the NCBI (https://www.ncbi.nlm.nih.gov/) website: RefSeq at Representative Genome Database, optimized for somewhat similar sequences, as general parameters: expect threshold 10,000, word size 7, as scoring parameters: match/mismatch 1, −3, without filters and without mask. The sequence showing the lowest identity percentage within the rest of the genome was chosen with particular attention at the 3′ end (seed region). The oligonucleotide pair chosen was sgRNAfw 5′-tcgaatactatgcttggatggcgg-3′ and sgRNArv 5′-aaacccgccatccaagcatagtat-3′ (GC% = 50).

### Preparation of the constructs

The target oligonucleotides (two μg each in 50 μl, 40 ng/μl) were annealed 10′ at 100 °C, cooled at RT, then diluted 20×. PtPuc3_diaCas9_sgRNA (https://www.addgene.org/109219/) has been digested with *Bsa*I and 50 ng of the linearized construct were ligated by T4 (2 h at RT) with four ng of insert. The ligation reaction was microdialized and two μl of it were electroporated in one shot top 10 cells (Invitrogen, Carlsbad, CA, USA). The clones were verified by digestion with *Bsa*I and *Hind*III (in positive clones the *Bsa*I restriction site is lost) and sequenced with pdiaCasfw 5′-CGGTGAGCTGGAAATTGGTT-3′and pdiaCasrv 5′-CCAAGACATGCTACCCGCA-3′ to verify the presence of the target sequence. The obtained plasmid pPtPuc3_diaCas9_sgRNA containing the target sequence (cargo vector) was chemically transformed in DH10B cells containing the conjugation vector pTA-Mob (Strand et al., 2014). Aliquots of transformed DH10B containing conjugation and cargo vectors were cryopreserved and inoculated the day before the conjugation for overnight growth.

### *P. tricornutum* conjugation with *E. coli*

Axenic CCMP632 strain of *P. tricornutum* Bohlin was obtained from the Provasoli-Guillard National Center for Culture of Marine Phytoplankton. Culture were grown in f/2 medium (Guillard, 1975) at 18 °C under white fluorescent lights (70 μmol m^−2^ s^−1^.), 12 h:12 h dark–light cycle. The DH10B containing pTA-Mob and pPtPuc3_diaCas9_sgRNA were used for the conjugation of *P. tricornutum* performed as in Karas et al. (2015) using f/2 as diatom medium as the only modification.

### Selection of resistant clones

Resistant clones were selected on ½f/2, 1% agarose, phleomycin 20 μg/ml containing plates. Colony PCR was performed as in De Riso et al. (2009) and PCR was performed on cell lysates with ShBlefw (5′-acgacgtgaccctgttcatc-3′)-ShBlerv (5′-gtcggtcagtcctgctcct-3′) and CEN6ARS4fw (5′-gaccacacacgaaaatcctg-3′)-HIS3rv (5′-gctctggaaagtgcctcatc-3′) primer pairs to ascertain the presence of the episome (pPtPuc3_diaCas9_sgRNA) in the cells.

### Sanger sequence analysis of the resistant clones

Resistant clones, resulting positive to the colony PCR amplification, were amplified by PCR with the locusfw (5′-atcctgtcgaaatggcaaaa-3′)-locusrv (5′-ttgtatcgctctacggcttg-3′) primer pair to produce and sequence the 403 bp amplicon encompassing the target locus.

### RT-PCR

Total RNA was extracted from *P. tricornutum* axenic cultures of wild type and mutant strains and cDNA retrotranscription was performed as in Russo et al. (2015). PCR was performed on cDNA with locusfw-locusrv primer pair to produce and sequence the 403 bp amplicon encompassing the target locus. PCR amplification was performed with the locusfw2 (5′-attggagaccattcgtgaag-3′)-locusrv (5′-ttgtatcgctctacggcttg-3′) primer pair to produce and sequence the 984 bp amplicon encompassing the target locus and containing single nucleotide polymorphisms (SNPs).

### *P. tricornutum* genomic DNA preparation and WGS

Genomic DNA was extracted from 400 ml wild type and mutant *P. tricornutum* axenic cultures in exponential growth phase by centrifugation, followed by freezing the cells in liquid nitrogen. The frozen pellet was then resuspended in two ml of lysis buffer, incubated at 37 °C for 15′ and centrifuged at 10,000×*g* at 4 °C for 10′. An equal volume of a mix of phenol:chloroform:isoamyl alcohol (25:24:1) was added to the supernatant and centrifuged for 15′. An equal volume of chloroform:isoamyl alcohol (24:1) was added to the supernatant and centrifuged in the same conditions. Two volumes of NaAc 3M pH 5.4 and ethanol 100% were added to the supernatant and precipitated at −20 °C. Following a centrifugation of 30′, five ml of ethanol 70% was added to the pellet twice to remove salts. The resulting pellet was resuspended in water and then treated with RNAse.

Genomic DNA libraries were prepared using KAPA Hyper Prep kit (PCR-free) following manufacturer’s standard protocol. Libraries have been sequenced by Illumina HiSeq 4000 as paired-end (2 × 150 bp) following manufacturer’s standard protocol. Data have been deposited in NCBI with the accession number PRJNA453101.

### WGS analyses

Raw sequencing reads were processed with FastQC (v0.11.3, http://www.bioinformatics.babraham.ac.uk/projects/fastqc/) and BBDuk (version 35.82, https://jgi.doe.gov/data-and-tools/bbtools/) to perform a quality check and to remove Illumina adapters and bases with a Phred-Like score less than 30. At the end of the quality check all the reads shorter than 35 bp were removed. High-quality reads were then mapped against the *P. tricornutum* reference genome (Ensembl Protists accession number ASM15095v2) with BWA MEM (version 0.7.12-r1039) (Li & Durbin, 2009) with default options. The obtained SAM files were then converted to BAM with samtools (v1.2) (Li et al., 2009) and then picard-tools (v1.131, https://broadinstitute.github.io/picard/index.html) was used to add read group and to remove optical duplicates. SNPs and small insertions or deletions (indels) were detected by processing the obtained BAM files with FreeBayes (v1.1.0-3-g961e5f3) (Garrison & Marth, 2012) with the following options: -m 30 -q 20 --min-coverage 5 --max-coverage 200 --genotype-qualities. The obtained VCF was filtered to keep only the variants with a quality score higher than 30 and with coverage in both the strains. Bigger structural variations were identified with the software Breakdancer (v1.4.5) (Chen et al., 2009). A manual curation was performed on the detected variants to focus only on those with a clearer difference between wild type and mutant. The heterozygosity rate across the genome was calculated as follows: the genome was divided in 50 kb windows with bedtools (Quinlan & Hall, 2010), then the windows were intersected with the Freebayes filtered VCF and the number of homozygous (1/1 or 0/0) and heterozygous (0/1) variants were calculated. Plots were generated with ggplot2 (Wickham, 2009) applying the loess smooth algorithm. The same procedure was followed to process public available Pt1 strain reads (SRX3566562).

Potential off-targets were predicted with the online Cas-OFFinder tool (http://www.rgenome.net/cas-offinder/new) (Bae, Park & Kim, 2014).

## Results

### Design of the target sequence

The coding sequence corresponding to the Phatr3_J46193 gene in *P. tricornutum* chr9: 533409–537647 locus, was scanned for the presence of NGG PAM sequences. Among the putative target sequences, the ones that occurred just upstream of the active sites of the protein were chosen. These sequences were submitted to a nucleotide BLAST analysis on the NCBI website against the *P. tricornutum* genome, and target sequences showing higher identity with sequences in other regions of the *P. tricornutum* genome were excluded. Particular attention, in the identity analysis, was paid to the 3′-end, because it has been proposed that the 8–12 PAM-proximal bases, known as the seed sequence, determine targeting specificity of the Cas9 protein (Nishimasu et al., 2014). No software for off-target prediction in diatoms were available at the beginning of this study. A target sequence with a GC content of 50% was chosen considering that it has been reported that a GC content higher than 70% may increase the probability of off-target effects (Tsai et al., 2015).

### Selection of mutant clones

The transformation efficiency was 2.0 × 10^−5^. One month after transformation we performed colony PCR on 14 clones to verify the presence of the episome in the cells, checking for the presence of two different regions of the episome, a fragment of the bleomycin antibiotic coding sequence and a fragment of the yeast centromeric CEN6-ARSH4-HIS3 sequence that enables episome maintenance in *P. tricornutum* (Karas et al., 2015) (Fig. S1). All the analyzed clones were successfully transformed. On a first group of 20 clones, resistant to the antibiotic selection, we performed colony PCR and sequenced 403 bp, encompassing the target sequence, obtaining two mutant clones. These displayed overlapping traces starting at the mutation site, an indication that two different sequences are present on the two alleles. After 1 month, we analyzed 20 additional clones with the same procedure and we found 16 mutated clones showing overlapping traces in chromatograms, observing an increase in the percentage of the mutated clones. The overlapping traces obtained in sequencing resulted from the presence of a mixture of PCR products that can be due to: (i) the occurrence of a monoallelic mutation (one allele is mutated and the other one is wild type), (ii) biallelic heterozygous mutations (both the alleles are mutated in different ways) or to (iii) a phenomenon of colony mosaicism, that is, a colony consists of a combination of cells with wild type and mutant alleles due to mutations occurring after transformed cells have started dividing (Daboussi et al., 2014).

**Figure 1:**
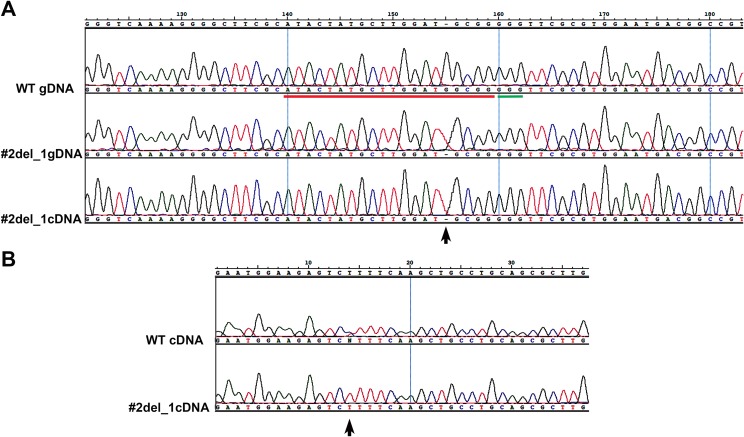
Single nucleotide deletion generated in transformant clone #2 and SNPs analysis of the target cDNA. (A) Chromatograms obtained sequencing the PCR performed on wild type genomic DNA, genomic DNA and cDNA of clone#2 del_1 cells. Sequence regions encompassing target site (red) and PAM (green) are aligned. Del_1 is indicated by a black arrow. (B) Chromatograms obtained from the PCR performed on cDNA of wild type and clone#2 del_1 cells. A region 676 bp upstream of the target region is shown. The position of one SNP is indicated by a black arrow.

We chose two clones and subcloned the PCR products in TOPO vector, obtaining, respectively, for clone#1: wild type, del_8, del_2, ins_7; for clone#2: del_13.1, del_23, del_13.2, del_33 (Fig. S2). We decided to focus on clone#2 by manually isolating 24 cells to obtain monoclonal cultures deriving from a single cell. From these 24 clones we obtained: one wild type, two clones with a del_1 with single peaks in the sequence (Fig. 1A), while the other 21 clones showed overlapping peaks indicating the occurrence of two different deletions on the two alleles or the presence of a monoallelic deletion together with a wild type allele (data not shown). We sequenced the same amplicon from the cDNA of the #2del_1 mutant clone and found the same mutation, confirming the presence of the single nucleotide deletion and excluding any repair events at the mRNA level (Fig. 1A).

### Isolation of a single clone with specific biallelic mutations

We decided to focus on the mutant clone#2 del_1 with the clear deletion of a single nucleotide. The single nucleotide deletion affected a guanine at position Chr9_535251, corresponding to position -4 immediately adjacent to the PAM sequence. This change in the transcript generated a frame-shift mutation causing the creation of a premature stop codon, and resulted in a truncated protein product lacking the active sites. The mutant strain was subcloned on plate and all of the resulting colonies displayed the same mutation, reinforcing the evidence that they derived from a single clone. Since all the sequences showed single peaks, we imagined two scenarios: the one nucleotide deletion was present on both the alleles (biallelic and homozygous mutation) or only this small mutation was visible by PCR and Sanger analysis and a deletion larger than the obtained amplicon was present (biallelic and heterozygous mutation). To distinguish between the two possibilities of a homozygous or a heterozygous mutation, we performed a PCR amplification on a larger cDNA fragment of 984 bp encompassing the locus of interest and containing SNPs. We obtained fragments of identical length from the wild type and from the mutant strain. Since SNPs were observed in the wild type sequence but not in the mutant (Fig. 1B), the presence of a large deletion on one of the alleles in the mutant could be hypothesized.

### Exclusion of Cas9 activity

Once we had identified the desirable mutation in the target locus, we eliminated the selective pressure of the antibiotic in the medium and repeated the colony PCR, verifying that the episome had been lost in the cells grown for 3 or 5 months in the absence of the antibiotic, while the episome was still present in the cells grown for 5 months under antibiotic pressure (Fig. S3).

### Analysis of off-target mutations in the genome by WGS

A wild type strain and a mutant strain in which the episome had been removed by relieving the selective pressure were sequenced by WGS. About 21 and 14 million of paired-end 150 nt reads were obtained from the wild type and the mutant strain, respectively. After trimming and duplicate removal, 10,237,553 and 16,194,883 of properly paired reads were mapped against the *P. tricornutum* genome from wild type and mutant, respectively, corresponding to a predicted coverage of 44× and 70×.

Mutant and wild type strains were compared to the reference genome and between themselves in order to identify both small variants (SNPs, indels) and larger structural variations, such as large insertions and deletions. Overall, 279,279 (81.31% SNPs) and 274,730 (81.32% SNPs) small variants were identified in mutant and wild type respect to the reference genome, respectively. Of those, 265,209 variants were identified both in the mutant and in the wild type, whereas 12,390 and 7,843 were found only in mutant and in the wild type, respectively. A total of 713 larger structural variations were identified comparing mutant and wild type with the reference genome, with a support of at least 10 reads. Those variants included 303 inter-chromosomal translocations, 294 deletions, 37 inversions and 79 intra-chromosomal translocations, with a mean variant size of 14,297 bp. When comparing wild type and mutant, we found seven variants specific for the mutant strain (one intra-chromosomal translocation and six deletions) and one deletion specific for the wild type (Table S1).

**Figure 2:**
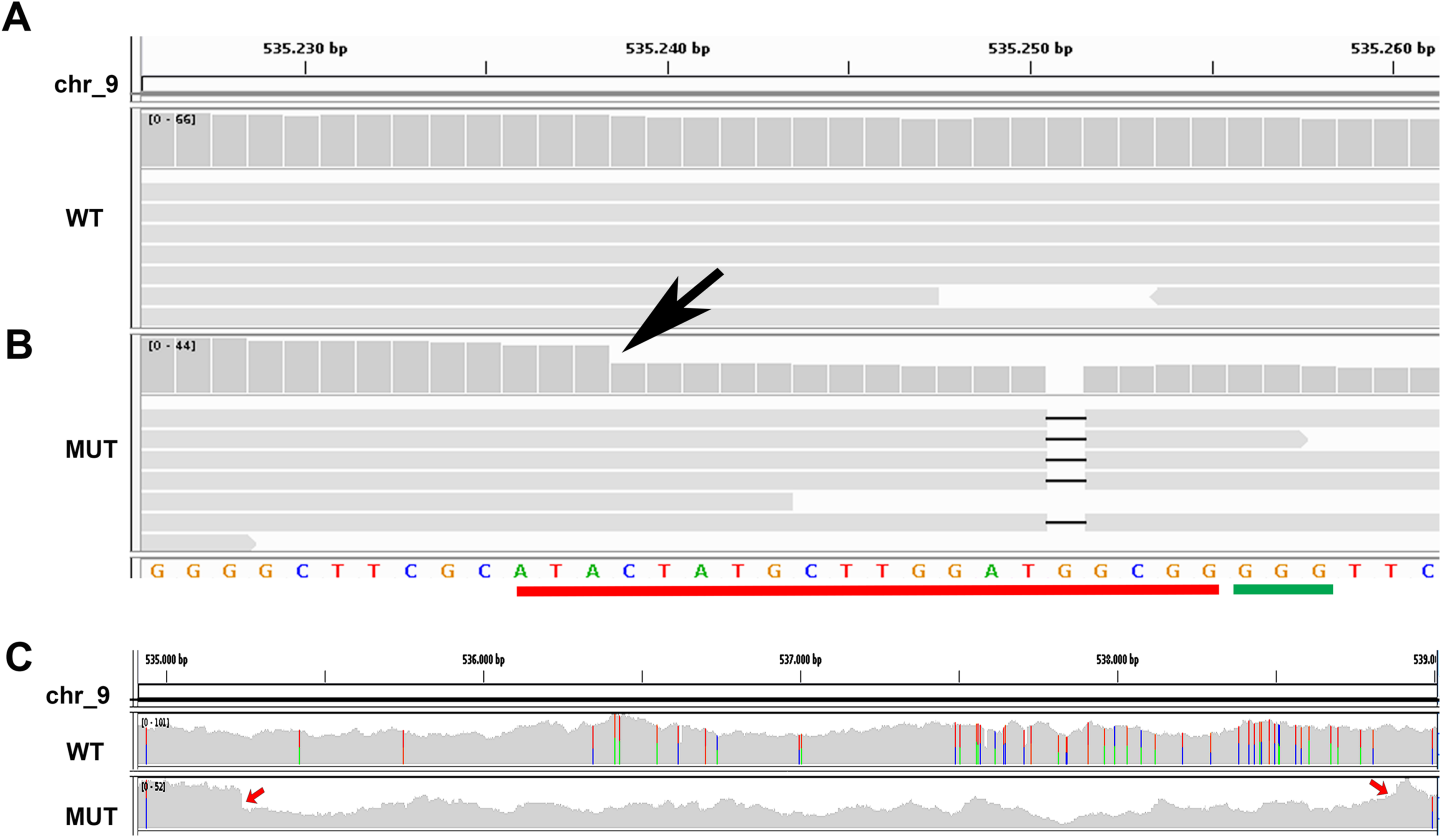
Whole genome resequencing analysis. (A and B) Integrative Genomics Viewer (IGV) visualization of a fragment of the Phatr3_gene on chromosome 9 encompassing the target sequence comparing the wild type Pt1 (WT) (A) and clone#2 del_1 (MUT) (B). Target site is outlined in red, the PAM in green. In the mutant the guanine deletion is clearly visible at position 535,251, and the starting point of a larger deletion corresponding to a halved number of the WGR read counts (gray vertical bars) can be observed starting from position 535,239 indicated by a black arrow. (C) IGV visualization of a larger region of chromosome 9 encompassing the entire large deletion of 3,639 bp. Colored bars are in correspondence of SNPs. In the mutant strain it is possible to see that there is a large deletion region delimited by red arrows and characterized by a decrease of the WGR read counts (gray peaks).

The results obtained from the variant calling successfully detected the mutant specific deletion of the G nucleotide (Chr9:535251) on one allele of the target Phatr3_J46193 locus (Figs. 2A and 2B). On the other allele in the same locus we detected a mutant-specific 3,639 nucleotides deletion (Chr9:535239–538878) (Fig. 2C) which was supported by the coverage reduction, the distance of the paired-ends and the presence of split-reads on the breaking point. This result confirmed our hypothesis of a large deletion on one of the alleles in the mutant (Fig. 1B).

By using the Cas-OFFinder tool we predicted 98 possible off-targets of Cas9, distributed on 15 chromosomes (Table S2). Interestingly none of the observed structural variations nor any specific SNP or indel were found in any of the potential off-targets in the mutant strain.

**Figure 3:**
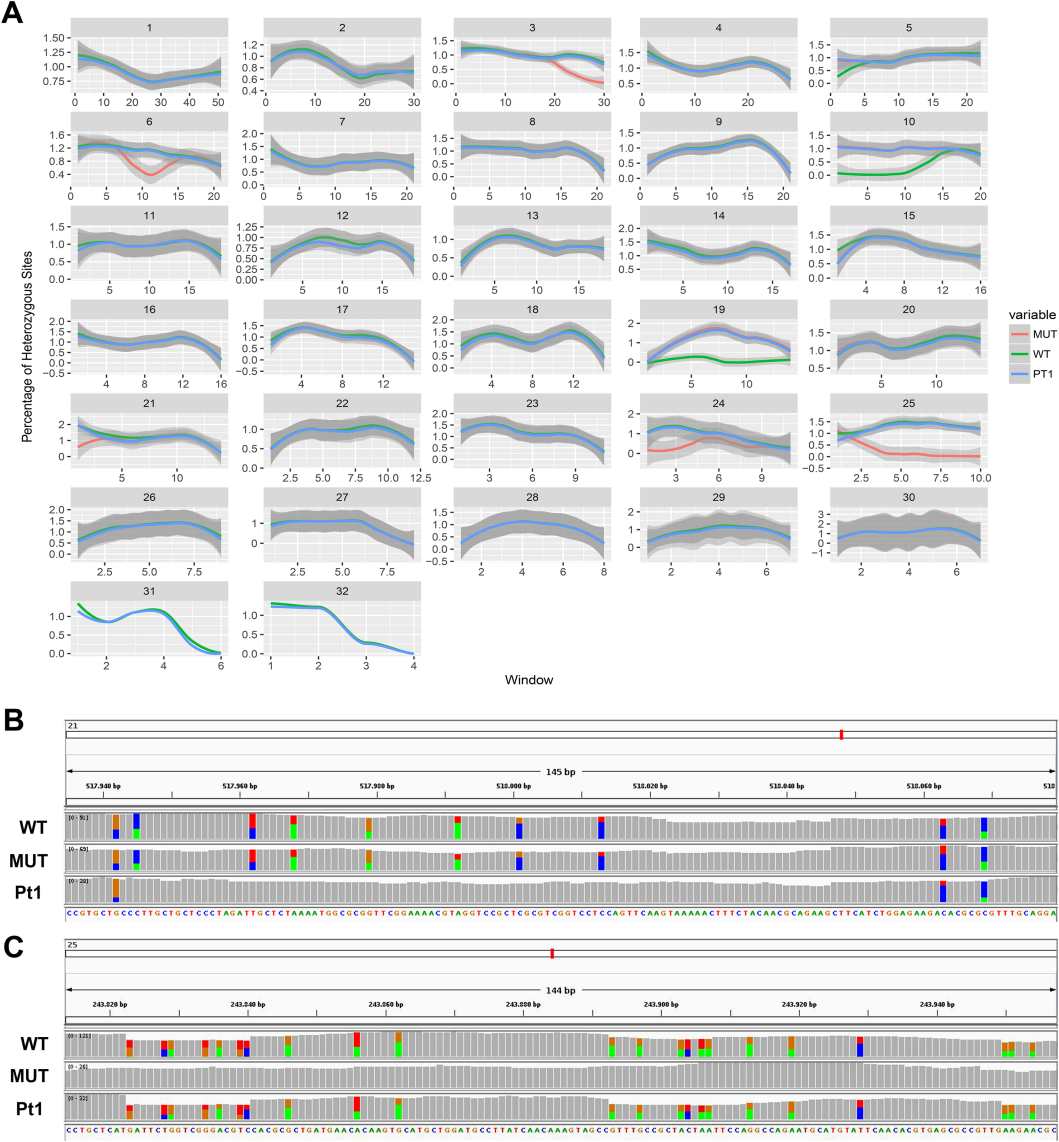
Loss of heterozygosity in wild type and mutant strains. (A) Genome-wide heterozygosity rate of WT (green line), MUT (orange line) and public Pt1 (blue line) strains. For each chromosome, the profile of the percentage of heterozygous polymorphisms is reported on the *Y*-axis calculated in 50 kb windows (*X*-axis). The gray smooth line indicates the 95% confidence level interval for predictions from a linear model, and was obtained applying a loess regression on the individual points. (B–C) Integrative Genomics Viewer (IGV) visualization of two examples of genomic regions characterized by LOH. Colored bars are in correspondence of SNPs. In (B) is shown a region on chromosome 21 in which the Pt1 strain is characterized by LOH (absence of SNPs compared with WT and MUT). In (C) the MUT strain showed LOH on chromosome 25.

On the other hand, we found long stretches of loss of heterozygosity (LOH) in the mutant clone respect to the wild type but also vice versa (Figs. 3A and 3B). More specifically, LOHs were observed for chromosomes 3, 6, 15, 21, 24 and 25 in the mutant, and for chromosomes 5, 10, 19 and 22 in the wild type. LOHs were very broad in size, ranging from a minimum of 20 kb (i.e. LOH on chromosome 15) to nearly an entire chromosome (LOH on chromosome 25). Some of the observed LOH included regions predicted as putative off-targets such as those on chromosomes 3, 6 and 25 (Table 1), and might be due to a break in one of the alleles successively repaired by homology-directed repair. However, no off-targets were predicted on the other chromosomes affected by LOH. It is important to point out that when LOH was observed this was not due to a deletion on one of the two chromosomes since the number of reads in that region remained as high as in the other regions (when a deletion occurs the number of mapped reads to that region is halved), therefore all the observed LOHs might be due to gene conversion.

**Table 1:** Levels of heterozygosity of LOH regions containing putative Cas9 off-targets.

Chromosome	Start	End	MUT HZ		WT HZ	
			Region (%)	Chromosome (%)	Region (%)	Chromosome (%)
3	1,065,985	1,448,384	0.09	0.83	1.10	1.10
6	455,938	603,852	0.04	0.95	1.24	1.12
25	119,890	497,000	0.06	0.31	1.30	1.31
Whole genome	–	–	0.98	–	0.98	–

**Notes:**

The percentage of heterozygosity has been calculated as the proportion of heterozygous variant sites over the region length.

WT, wild type; MUT, mutant; HZ, heterozygosity.

In order to get more insights into the observed variation in heterozygosity, a public dataset of WGS of the Pt1 strain, hereafter named “public Pt1” (Rastogi et al., 2017) was processed with the same bioinformatics pipeline. The results in Figs. 3A and 3B highlight that the three strains show very similar profiles of heterozygosity across the genome and the only variations affect either the mutant or the wild type strains. Interestingly, the Pearson correlation of the allele frequencies of the observed variants of wild type and mutant with the public Pt1 was higher (0.78) then the correlation between wild type and mutant (0.61). Besides, the similarity of Pt1, wild type and mutant strain was evaluated by calculating Pearson Correlation and Euclidean distance scores using all the detected variants (Table 2). Both the measures highlighted that the WT strain is more similar to Pt1 than the mutant strain.

**Table 2:** Measures of similarity and distance among the strains.

Pearson correlation/Euclidean distance	MUT	WT	Pt1
MUT		353.31	318.31
WT	0.29		244.80
Pt1	0.35	0.40	

**Note:**

Pearson correlation (decimal numbers, lower triangle) and Euclidean distance (three-figure numbers, upper triangle) between MUT, WT and Pt1 strains. The scores were calculated after converting the genotype information to scalar numbers.

## Discussion

The CRISPR/Cas9 system has been used for genome editing in a wide-ranging group of organisms, from microbes to human, and it has been observed that the efficiency and mutation quality depends on a number of factors including target site choice, sgRNA design, the properties of the nuclease, the quantity of nuclease and sgRNA, and the intrinsic differences in DNA repair pathways in the diverse species (Lee et al., 2016; Miyaoka et al., 2016; Bothmer et al., 2017; Xu, Duan & Chen, 2017; Shin et al., 2017; Cebrian-Serrano & Davies, 2017). Species-dependent effects include the prevalence of NHEJ compared to homology directed recombination (HDR) in higher eukaryotes and subtle differences in the mutation signatures generated in animals and plants. While CRISPR/Cas9 mainly introduces small deletions (<10 bp) and single-base insertions in plants, both types of indels tend to be larger (deletions <40 bp and insertions of 1–15 bp) and there is a greater frequency of larger deletions in animals (Bortesi et al., 2016; Kapusi et al., 2017; Shin et al., 2017). In addition, while bacteria have a preference for HDR, eukaryotic microbes showed diverse reactions when exposed to CRISPR/Cas9. For example, Cas9 expression was toxic in *Chlamydomonas reinhardtii* when constitutively expressed, while transient expression of the protein along with sgRNA was able to induce indels by NHEJ (Jiang et al., 2014). On the contrary, *Plasmodium falciparum* showed to be deficient in the NHEJ pathway but not in the HDR when a donor template was provided (Ghorbal et al., 2014).

We applied the CRISPR/Cas9 technology in *P. tricornutum* obtaining different indel mutations at the target site. We used genomic PCR and Sanger sequencing as screening procedure to evaluate the efficiency of the method and the quality of mutations obtained. We also observed an increment of the proportion of the mutant cells by extending the cultivation period, probably reflecting the accumulation of new mutations (Mikami, Toki & Endo, 2015, 2016).

While previous studies in diatoms reported predominantly identical biallelic mutation consisting mostly in <40 bp deletions, with the exception of one 212 bp insertion (Nymark et al., 2016; Hopes et al., 2016), we obtained biallelic heterozygous mutations on the two alleles or monoallelic mutations as indicated by the overlapping peaks in the sequences chromatograms of the isolated cells, mostly shorter than 40 bp, but we also obtained a deletion larger than 3,500 bp, that was not detectable by PCR-based methods and was revealed by WGS analysis (Fig. 2). In the selected clone with this large deletion on one allele and a single nucleotide deletion on the other, we expect absence of a functional protein since the single nucleotide mutation leads to a frame-shift and a premature stop codon, resulting in a truncated protein product lacking the active sites. In the mutant clone, we observed only a mild phenotype characterized by a moderate decrease in growth (data not shown) probably because the cell is able to activate a compensation mechanism. More accurate phenotypic analyses will be performed in future studies, while the resequencing data presented in this study allowed to explore the extent of putative off-target effects of Cas9 and of genomic changes in the mutant strain.

As widely reported in literature, the frequency of off-target mutations depends on the abundance of the Cas9/sgRNA complex so the off-target probability can be reduced by transient expression of the components rather than stable transgene integration in the genome (Tsai et al., 2015). For this reason, we coupled the CRISPR/Cas9 technology with bacterial conjugation: the transgene in the form of episome permits to exclude the nuclease activity when it is no more required, and moreover avoids the occurrence of unwanted perturbation of gene expression due to the position effect of the transgene in the genome. A low frequency of unwanted mutations has been reported when the sgRNA features mismatches outside the seed sequence, for example in rice (Zhang, Wen & Guo, 2014; Xu et al., 2015) and wheat (Upadhyay et al., 2013), indicating that such events could be avoided by designing more specific sgRNAs. Therefore, selecting sites predicted to have the most specific seed regions with the fewest possible off-target mismatches may be crucial to improving on-target efficiency. In contrast, the PAM distal sequence has been suggested to be less important for specificity, and mismatches in this region are more likely to be tolerated (Liu et al., 2016). The choice of a target sequences with a GC content lower than 70% also allows to decrease the likelihood of off-target effects (Tsai et al., 2015).

Off-target mutations in human cells were reported by some authors to be more common than mutations at the on-target site, raising concerns about the intrinsic fidelity of the CRISPR/Cas9 system (Fu et al., 2013; Mali et al., 2013; Pattanayak et al., 2013; Hsu, Lander & Zhang, 2014; Kim et al., 2015). Indeed, this was because many studies were conducted on cancer cell lines, which present often dysfunctional repair mechanisms. In fact, when stem cells were used, no off-targets or very few events were reported (Smith et al., 2014; Veres et al., 2014). Negligible off-target effects activity was reported in zebrafish (Hruscha et al., 2013), chicken (Oishi et al., 2016) and rabbit (Lv et al., 2016). However, it is important to stress that most studies have analyzed off-targets at predicted sites rather than by WGS and in microorganisms unspecific mutations have not been widely investigated (Bortesi et al., 2016). In a recent work, which represents the only report on this topic for diatoms, Stukenberg et al. (2018) showed that using target RNAs predicted to have a high off-target score did not result in off-target mutations at the prediction sites. Since they used a PCR-based approach and only examined two potential off-target sites in five different strains, they could not exclude the possibility that the CRISPR/Cas9 system interfered with other genomic regions.

By performing WGS, we were able to reveal a very large deletion at the target site on one of the two alleles. Interestingly, we found a substantial number of mutations and also very large gene conversion phenomena in multiple regions of the genome in the mutant but also in the wild type grown for our experiments, and in a wild type strain grown in a different laboratory (Fig. 3A). We point out that the mutant strain and the wild type strain used in our study have been collected for sequencing almost 1 year after they were separated, and during this time have accumulated differences that are therefore most likely due to an in-culture effect, which could be more pronounced than the Cas9 off-target effects themselves. It is thus difficult to establish how many of the changes observed in the mutant could be ascribed to the effect of the nuclease activity and additional dedicated analyses will be required. A larger set of samples and additional controls, that is, episomes containing Cas9 only or sgRNA only, and multiple genomic targets for Cas9, will increase the amount of information and will allow to detect possible, if any, preferential off-target sites of the Cas9 nuclease. Resequencing of additional wild type strains from different laboratories could also shed light on the extent of rearrangements in cultures maintained for long periods.

## Conclusions

In our study, Cas9 delivery through bacterial conjugation allowed to minimize the time of exposure of the genome to the nuclease activity.

Through WGS, we detected genomic changes, mainly large regions showing LOH, in the mutant as well as in wild type strain. This can likely be ascribed to the effect of prolonged cultivation. The occurrence of genome changes due to cultivation has to be taken into account when comparing wild type and mutant and also when comparing data from different laboratories in which *P. tricornutum* cultures have been grown and evolved independently. Cryopreservation of mutants as soon as they are obtained, and of the wild type original culture, could reduce the cultivation bias in the phenotypic analyses to be performed downstream.

Our results represent the proof of concept that the technology can be safely applied to diatoms, opening new unexplored opportunities to exploit the unlimited resources of this group of organisms that are precious from both ecological and biotechnological viewpoints.

## Supplemental Information

10.7717/peerj.5507/supp-1 Supplemental Information 1 Fig. S1. Screening of transformant clones. PCR analysis of 14 resistant clones (lanes 1-14), a bleomycin resistant clone (lane 15), Pt1 wild type strain (lane 16) with primer pairs amplifying the ShBle cassette (upper panel) and the yeast CEN6-ARSH4-HIS3 region (lower panel). B, blank. The 100 bp ladder is displayed on the right.

10.7717/peerj.5507/supp-2 Supplemental Information 2 Fig. S2. InDels generated in two transformant clones. InDels detected by subcloning in the TOPO vector the PCR fragments obtained for clones #1 and #2. The sequence corresponding to the target site is underlined in red, PAM in green, clone#1 insertion is underlined in black.

10.7717/peerj.5507/supp-3 Supplemental Information 3 Fig. S3. Cas9 activity exclusion from the cells. PCR analysis on clone#2 del_1 and wild type Pt1 grown for 3 months (lanes 1 and 2) and for 5 months (lanes 3 and 4) without antibiotic selection, clone#2 del_1 grown for 5 months with antibiotic selective pressure (lane 5) with primer pairs amplifying the ShBle cassette (upper panel) and, as a positive control (ctrl), a region encompassing the target locus (lower panel). B, blank, 100, 100 bp ladder.

10.7717/peerj.5507/supp-4 Supplemental Information 4 Table S1. Strain specific variants. Type, size and positions of the variants observed in WT and MUT are reported. The last column indicates if the region was predicted as a potential off-target.

10.7717/peerj.5507/supp-5 Supplemental Information 5 Table S2. List of potential off-targets predicted by Cas-OFFinder tool. For each potential site, the coordinates, the reference sequence and observed variants are reported. In addition, the percentage of supporting reads is indicated together with the associated locus.

10.7717/peerj.5507/supp-6 Supplemental Information 6 Full-length uncropped gel Fig. S1. Refer to the lanes 3-17 for both top and bottom panels.

10.7717/peerj.5507/supp-7 Supplemental Information 7 Full-length uncropped gel Fig. S3.

## Additional Information and Declarations

### Data Availability

The following information was supplied regarding data availability: Data have been deposited in NCBI with the accession number PRJNA453101.
