# Supplementary figures and images for: Assessment of genomic changes in a CRISPR/Cas9 Phaeodactylum tricornutum mutant through whole genome resequencing
Source: PeerJ. 2018 Oct 5;6:e5507. doi: 10.7717/peerj.5507 (PMC6174884; doi:10.7717/peerj.5507)

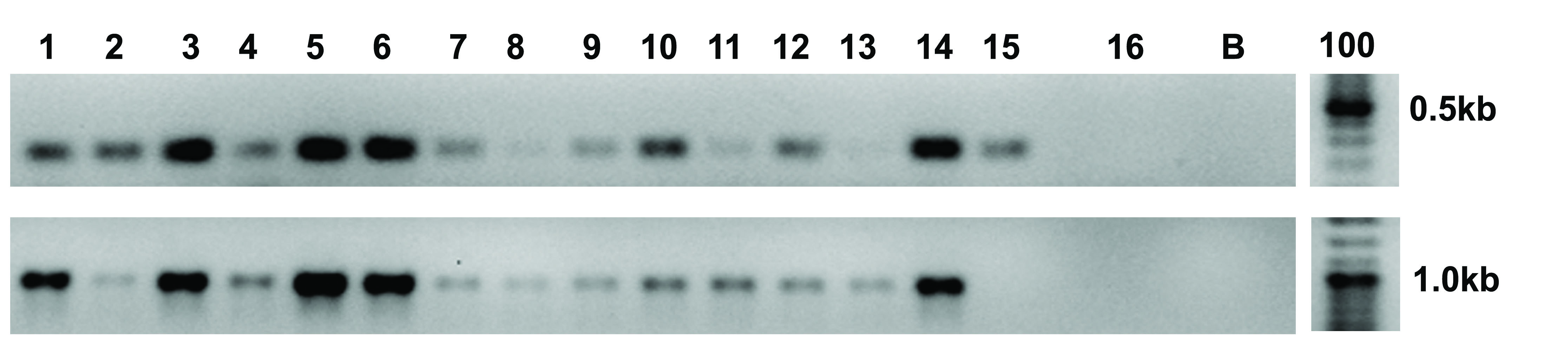

Supplement: Supplemental Information 1 — PCR analysis of 14 resistant clones (lanes 1-14), a bleomycin resistant clone (lane 15), Pt1 wild type strain (lane 16) with primer pairs amplifying the ShBle cassette (upper panel) and the yeast CEN6-ARSH4-HIS3 region (lower panel). B, blank. The 100 bp ladder is displayed on the right. [file peerj-06-5507-s001.jpg]

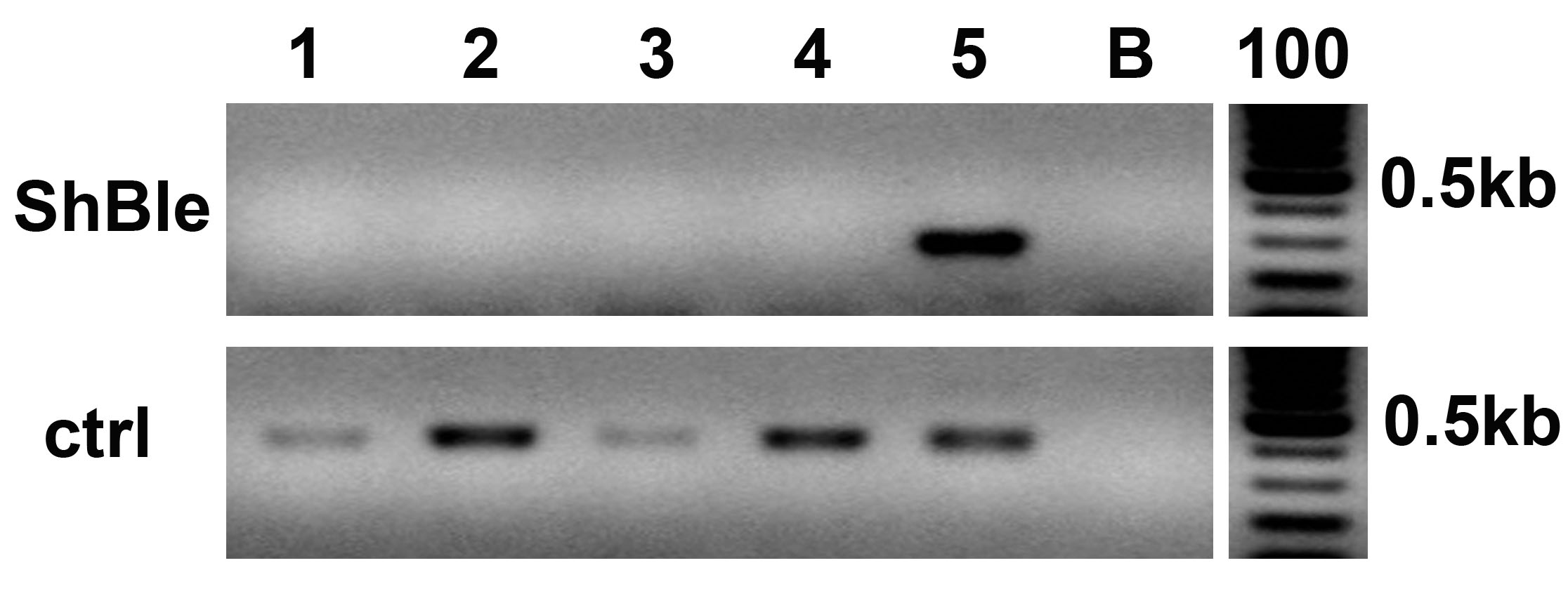

Supplement: Supplemental Information 3 — PCR analysis on clone#2 del_1 and wild type Pt1 grown for 3 months (lanes 1 and 2) and for 5 months (lanes 3 and 4) without antibiotic selection, clone#2 del_1 grown for 5 months with antibiotic selective pressure (lane 5) with primer pairs amplifying the ShBle cassette (upper panel) and, as a positive control (ctrl), a region encompassing the target locus (lower panel). B, blank, 100, 100 bp ladder. [file peerj-06-5507-s003.jpg]

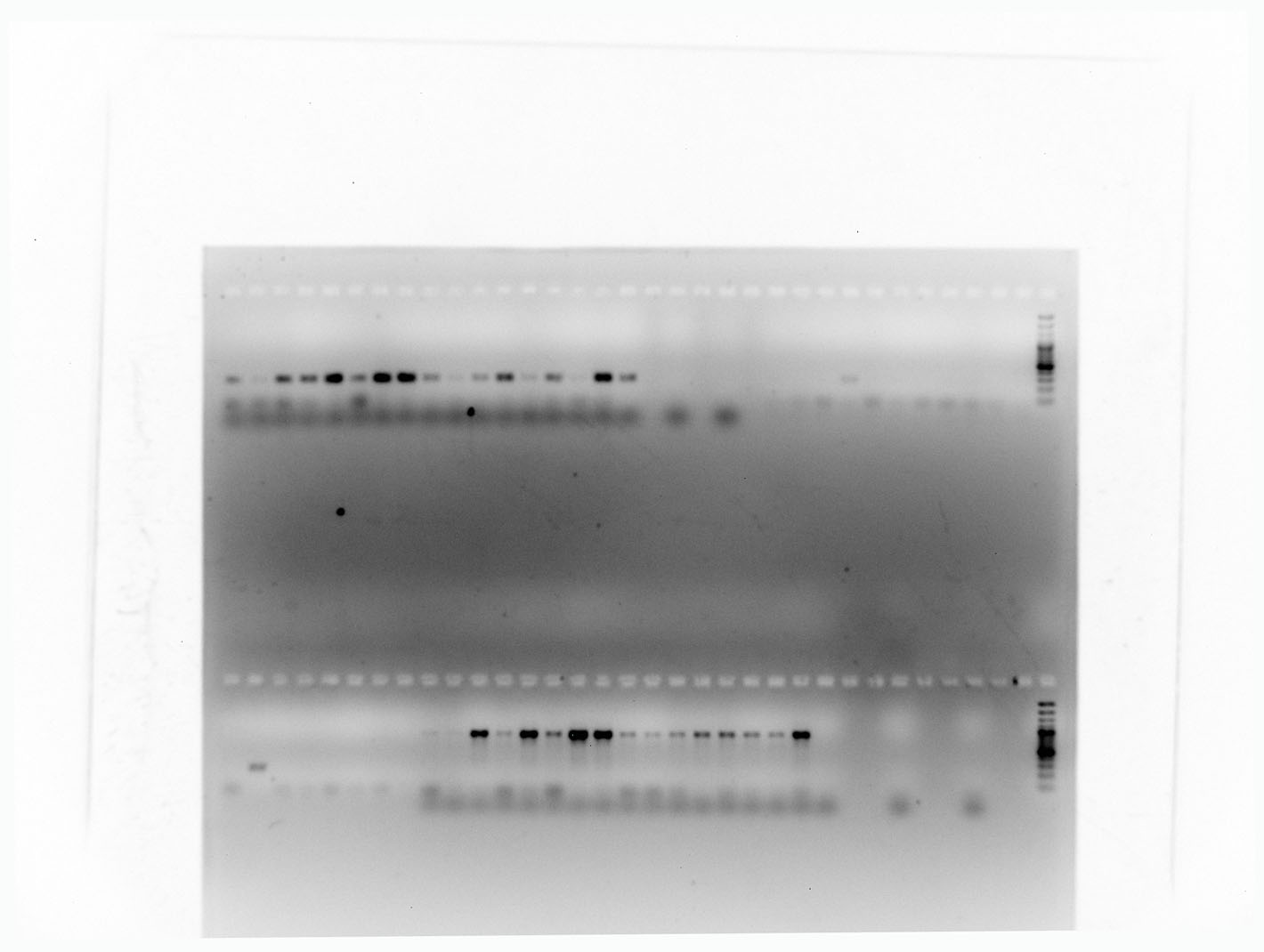

Supplement: Supplemental Information 6 — Refer to the lanes 3-17 for both top and bottom panels. [file peerj-06-5507-s006.jpg]

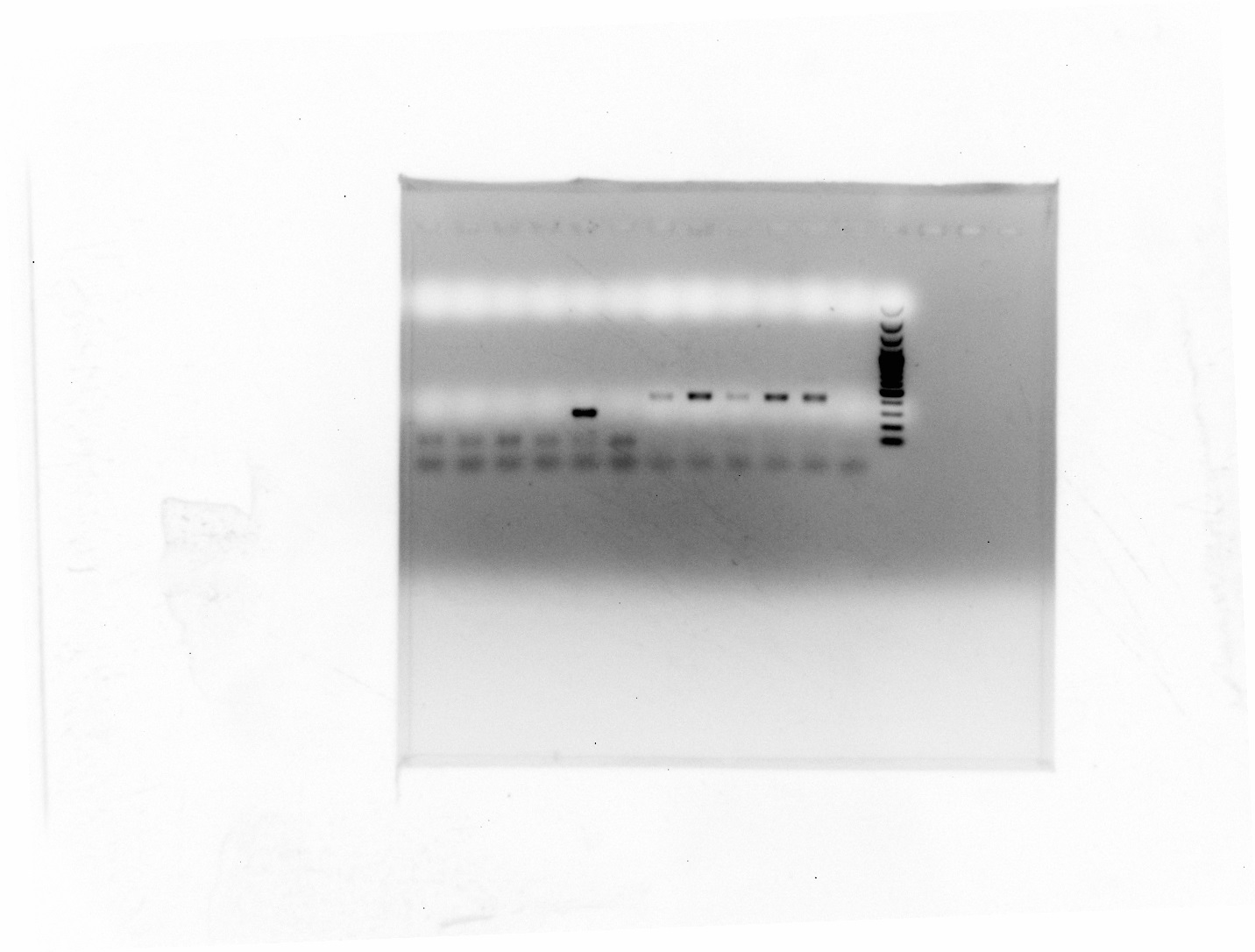

Supplement: Supplemental Information 7 [file peerj-06-5507-s007.jpg]
