# Supplementary material for: Assessment of genomic changes in a CRISPR/Cas9 Phaeodactylum tricornutum mutant through whole genome resequencing
Source: PeerJ. 2018 Oct 5;6:e5507. doi: 10.7717/peerj.5507 (PMC6174884; doi:10.7717/peerj.5507)
Supplement: Supplemental Information 4 — Type, size and positions of the variants observed in WT and MUT are reported. The last column indicates if the region was predicted as a potential off-target. [file peerj-06-5507-s004.docx]

| **Chromosome** | **Strain** | **Start** | **End** | **Type** | **Size** | **Potential OffTarget?** |
| --- | --- | --- | --- | --- | --- | --- |
| 3 | WT | 1385674 | 1387573 | DEL | 1899 | No |
| 7 | MUT | 121164 | 123927 | DEL | 2763 | No |
| 10 | MUT | 19591 | 23085 | Intrachromosomal Translocation | 3494 | No |
| 10 | MUT | 561290 | 561855 | DEL | 565 | No |
| 19 | MUT | 374544 | 381065 | DEL | 6521 | No |
| 19 | MUT | 413983 | 414573 | DEL | 590 | No |
| 21 | MUT | 201885 | 226956 | DEL | 25071 | No |
