# Supplementary material for: Assessment of genomic changes in a CRISPR/Cas9 Phaeodactylum tricornutum mutant through whole genome resequencing
Source: PeerJ. 2018 Oct 5;6:e5507. doi: 10.7717/peerj.5507 (PMC6174884; doi:10.7717/peerj.5507)
Supplement: Supplemental Information 5 — For each potential site, the coordinates, the reference sequence and observed variants are reported. In addition, the percentage of supporting reads is indicated together with the associated locus. [file peerj-06-5507-s005.docx]

| **Chromosome** | **Start** | **End** | **Reference** | **Alternative** | **%MUT reads** | **%WT reads** | **Locus** |
| --- | --- | --- | --- | --- | --- | --- | --- |
| 9 | 535250 | 535251 | TGG | TG | 100,00 | 0,00 | Phatr3_J46193 |
| 12 | 658146 | 658147 | C | A | 21,05 | 3,45 | Phatr3_J37297 |
| 25 | 130666 | 130667 | G | A | 100,00 | 42,37 | chromosome:25 |
| 3 | 1204407 | 1204408 | T | C | 100,00 | 51,56 | chromosome:3 |
| 3 | 1204436 | 1204437 | C | T | 100,00 | 52,54 | Phatr3_J44174 |
| 2 | 102791 | 102792 | A | G | 53,57 | 28,57 | Phatr3_J9947 |
| 25 | 131014 | 131015 | T | G | 100,00 | 54,24 | chromosome:25 |
| 2 | 102990 | 102991 | A | T | 53,85 | 34,15 | Phatr3_J9947 |
| 25 | 107108 | 107109 | G | A | 70,37 | 45,00 | Phatr3_EG02222 |
| 25 | 107108 | 107109 | G | A | 70,37 | 45,00 | Phatr3_EG00216 |
| 17 | 563146 | 563147 | A | T | 57,45 | 36,99 | chromosome:17 |
| 2 | 860168 | 860169 | C | T | 71,79 | 47,06 | Phatr3_J32815 |
| 25 | 107198 | 107199 | T | C | 78,95 | 51,79 | Phatr3_EG02222 |
| 25 | 107198 | 107199 | T | C | 78,95 | 51,79 | Phatr3_EG00216 |
| 2 | 860147 | 860148 | G | C | 66,67 | 45,07 | Phatr3_J32815 |
| 25 | 107014 | 107015 | G | A | 77,42 | 52,38 | Phatr3_EG02222 |
| 25 | 107014 | 107015 | G | A | 77,42 | 52,38 | Phatr3_EG00216 |
| 6 | 668668 | 668669 | A | G | 54,05 | 36,67 | chromosome:6 |
| 25 | 106871 | 106872 | CCT | TCG | 73,68 | 50,00 | Phatr3_EG02222 |
| 25 | 106871 | 106872 | CCT | TCG | 73,68 | 50,00 | Phatr3_EG00216 |
| 30 | 195377 | 195378 | C | T | 69,57 | 47,83 | Phatr3_J41450 |
| 17 | 563113 | 563114 | ATG | AG | 56,36 | 38,89 | chromosome:17 |
| 2 | 859844 | 859845 | G | A | 62,79 | 45,16 | Phatr3_J32815 |
| 17 | 563159 | 563160 | A | T | 52,08 | 37,68 | chromosome:17 |
| 17 | 563202 | 563203 | T | C | 45,95 | 33,33 | chromosome:17 |
| 2 | 859964 | 859965 | A | T | 64,00 | 47,14 | Phatr3_J32815 |
| 6 | 668615 | 668616 | A | C | 52,63 | 38,81 | chromosome:6 |
| 1 | 1742550 | 1742551 | G | A | 62,07 | 47,14 | Phatr3_J42998 |
| 2 | 859874 | 859875 | C | A | 64,44 | 49,25 | Phatr3_J32815 |
| 30 | 195150 | 195151 | T | A | 68,35 | 53,19 | chromosome:30 |
| 2 | 860078 | 860079 | T | C | 58,97 | 45,98 | Phatr3_J32815 |
| 25 | 106835 | 106836 | C | T | 68,00 | 53,85 | Phatr3_EG02222 |
| 25 | 106835 | 106836 | C | T | 68,00 | 53,85 | Phatr3_EG00216 |
| 13 | 140820 | 140821 | T | C | 61,70 | 50,51 | chromosome:13 |
| 30 | 195274 | 195275 | T | C | 72,13 | 59,09 | chromosome:30 |
| 17 | 563476 | 563477 | G | A | 52,50 | 43,10 | Phatr3_J15019 |
| 30 | 195107 | 195108 | G | C | 69,33 | 57,05 | chromosome:30 |
| 6 | 998740 | 998741 | G | A | 62,96 | 51,85 | Phatr3_J35067 |
| 1 | 1742457 | 1742458 | A | G | 66,67 | 54,93 | Phatr3_J42998 |
| 30 | 195077 | 195078 | A | G | 68,57 | 58,78 | chromosome:30 |
| 17 | 563417 | 563418 | A | G | 50,00 | 43,84 | Phatr3_J15019 |
| 6 | 668547 | 668548 | A | G | 55,26 | 49,28 | Phatr3_J34923 |
| 6 | 668370 | 668371 | CA | TC | 50,00 | 46,03 | Phatr3_J34923 |
| 7 | 905092 | 905093 | G | T | 59,26 | 55,26 | Phatr3_J45677 |
| 7 | 905117 | 905118 | C | G | 53,33 | 50,00 | Phatr3_J45677 |
| 20 | 269953 | 269954 | C | T | 55,81 | 53,03 | Phatr3_J54974 |
| 17 | 563403 | 563404 | G | A | 46,00 | 43,75 | chromosome:17 |
| 6 | 668405 | 668406 | T | G | 51,52 | 50,00 | Phatr3_J34923 |
| 20 | 270002 | 270003 | T | A | 58,14 | 56,45 | Phatr3_J54974 |
| 7 | 905098 | 905099 | C | T | 46,43 | 45,68 | Phatr3_J45677 |
| 2 | 860047 | 860048 | G | A | 48,57 | 48,05 | Phatr3_J32815 |
| 7 | 905112 | 905113 | G | A | 51,72 | 51,22 | Phatr3_J45677 |
| 17 | 563388 | 563389 | T | C | 46,00 | 45,68 | chromosome:17 |
| 16 | 611676 | 611677 | A | G | 57,50 | 57,38 | Phatr3_EG02280 |
| 7 | 905052 | 905053 | C | A | 54,55 | 54,43 | Phatr3_J45677 |
| 17 | 563298 | 563299 | AT | ACAGT | 96,88 | 96,97 | chromosome:17 |
| 6 | 998573 | 998574 | C | T | 44,44 | 45,10 | Phatr3_J35067 |
| 11 | 645543 | 645544 | C | A | 51,35 | 52,78 | Phatr3_J36913 |
| 6 | 668503 | 668504 | A | G | 55,17 | 56,72 | Phatr3_J34923 |
| 18 | 66396 | 66397 | A | G | 51,61 | 53,70 | Phatr3_J22510 |
| 18 | 66633 | 66634 | T | C | 53,66 | 56,52 | Phatr3_J22510 |
| 18 | 66832 | 66833 | A | C | 47,06 | 50,00 | Phatr3_J22510 |
| 6 | 668401 | 668402 | G | A | 47,06 | 50,00 | Phatr3_J34923 |
| 18 | 66823 | 66824 | C | A | 45,45 | 48,48 | Phatr3_J22510 |
| 1 | 1742625 | 1742626 | T | C | 45,95 | 50,00 | Phatr3_J42998 |
| 17 | 563326 | 563327 | A | T | 43,55 | 47,95 | chromosome:17 |
| 18 | 35966 | 35967 | T | G | 42,86 | 47,54 | Phatr3_EG00468 |
| 18 | 66578 | 66579 | T | A | 43,59 | 48,98 | Phatr3_J22510 |
| 6 | 668650 | 668651 | T | C | 51,35 | 59,32 | chromosome:6 |
| 18 | 36011 | 36012 | C | A | 36,36 | 43,08 | Phatr3_EG00468 |
| 16 | 611606 | 611607 | T | C | 55,00 | 65,38 | Phatr3_EG02280 |
| 6 | 998450 | 998451 | G | C | 46,81 | 57,63 | Phatr3_J35067 |
| 18 | 66510 | 66511 | A | G | 47,22 | 58,73 | Phatr3_J22510 |
| 18 | 66489 | 66490 | A | T | 44,44 | 55,74 | Phatr3_J22510 |
| 18 | 66555 | 66556 | T | C | 46,51 | 59,32 | Phatr3_J22510 |
| 17 | 563212 | 563213 | C | T | 51,22 | 67,27 | chromosome:17 |
| 7 | 904829 | 904830 | T | C | 42,86 | 56,47 | Phatr3_J45677 |
| 7 | 904850 | 904851 | G | A | 41,03 | 55,13 | Phatr3_J45677 |
| 30 | 195085 | 195086 | G | A | 30,56 | 42,36 | chromosome:30 |
| 13 | 140528 | 140529 | C | T | 31,82 | 44,29 | Phatr3_EG00868 |
| 17 | 563228 | 563229 | G | A | 44,23 | 62,71 | chromosome:17 |
| 17 | 563291 | 563292 | GAA | GA | 34,62 | 49,23 | chromosome:17 |
| 18 | 66478 | 66479 | GCAAT | GCAACAAGAACAAC | 35,29 | 51,02 | Phatr3_J22510 |
| 18 | 66696 | 66697 | A | C | 39,39 | 57,45 | Phatr3_J22510 |
| 30 | 195126 | 195127 | A | C | 28,57 | 41,78 | chromosome:30 |
| 2 | 103098 | 103099 | G | A | 38,71 | 60,98 | chromosome:2 |
| 1 | 698443 | 698444 | C | T | 40,54 | 67,69 | Phatr3_EG01814 |
| 6 | 668796 | 668797 | A | C | 41,46 | 70,87 | chromosome:6 |
| 12 | 658134 | 658135 | C | T | 11,76 | 20,69 | Phatr3_J37297 |
| 30 | 195293 | 195294 | A | G | 23,44 | 42,28 | Phatr3_J41450 |
| 25 | 130953 | 130954 | A | G | 0,00 | 52,63 | chromosome:25 |
| 3 | 1204237 | 1204238 | G | T | 0,00 | 48,48 | chromosome:3 |
| 3 | 1204248 | 1204249 | A | G | 0,00 | 53,13 | chromosome:3 |
| 3 | 1204333 | 1204334 | C | T | 0,00 | 55,36 | chromosome:3 |
| 3 | 1204470 | 1204471 | TC | TCAC | 0,00 | 80,95 | Phatr3_J44174 |
| 3 | 1204475 | 1204476 | CC | CATC | 0,00 | 39,58 | Phatr3_J44174 |
| 3 | 1204587 | 1204588 | A | G | 0,00 | 36,17 | Phatr3_J44174 |
| 9 | 535421 | 535422 | A | T | 0,00 | 49,28 | Phatr3_J46193 |
